# Narrative Review of *n*-3 Polyunsaturated Fatty Acid Supplementation upon Immune Functions, Resolution Molecules and Lipid Peroxidation

**DOI:** 10.3390/nu13020662

**Published:** 2021-02-18

**Authors:** Gary P. Zaloga

**Affiliations:** MedSciHealth Consultants, 12931 Sorrento Way, Bradenton, FL 34211, USA; gpzaloga@aol.com

**Keywords:** *n*-3 polyunsaturated fatty acids, fish oil, docosahexaenoic acid, eicosapentaenoic acid, resolution molecules, resolvins, peroxidation, vitamin E, immune response, inflammation

## Abstract

Fish oil supplementation is commonplace in human nutrition and is being used in both enteral and parenteral formulations during the treatment of patients with a large variety of diseases and immune status. The biological effects of fish oil are believed to result from their content of *n*-3 polyunsaturated fatty acids (PUFA), particularly docosahexaenoic acid (DHA) and eicosapentaenoic acid (EPA). These fatty acids are known to have numerous effects upon immune functions and are described as immunomodulatory. However, immunomodulatory is a nondescript term that encompasses immunostimulation and immunosuppression. The primary goal of this review is to better describe the immune effects of *n*-3 PUFA as they relate to immunostimulatory vs. immunosuppressive effects. One mechanism proposed for the immune effects of *n*-3 PUFA relates to the production of specialized pro-resolving mediators (SPMs). A second goal of this review is to evaluate the effects of *n*-3 PUFA supplementation upon production of SPMs. Although *n*-3 PUFA are stated to possess anti-oxidative properties, these molecules are highly oxidizable due to multiple double bonds and may increase oxidative stress. Thus, the third goal of this review is to evaluate the effects of *n*-3 PUFA upon lipid oxidation. We conclude, based upon current scientific evidence, that *n*-3 PUFA suppress inflammatory responses and most cellular immune responses such as chemotaxis, transmigration, antigen presentation, and lymphocyte functions and should be considered immunosuppressive. *n*-3 PUFA induced production of resolution molecules is inconsistent with many resolution molecules failing to respond to *n*-3 PUFA supplementation. *n*-3 PUFA supplementation is associated with increased lipid peroxidation in most studies. Vitamin E co-administration is unreliable for prevention of the lipid peroxidation. These effects should be considered when administering *n*-3 PUFA to patients that may be immunosuppressed or under high oxidative stress due to illness or other treatments.

## 1. Introduction

Fatty acids are important components of the human diet, providing a source of energy and essential fatty acids (i.e., indispensable fatty acids that cannot be synthesized by human cells). Fatty acids provide structure and modulate functions of cell membranes, act as cellular messengers in signal-transduction pathways, serve as mediators and regulators of immune functions, and are components of lipid transport particles such as chylomicrons and lipoproteins. Fatty acids may be grouped into different categories based upon carbon chain length (i.e., short chain 2–4 carbons, medium chain 6–12 carbons, long chain 14 or more carbons), number of double bonds (i.e., saturated—no double bonds, monounsaturated—1 double bond, polyunsaturated—2 or more double bonds), and location of the first double bond from the methyl end of the molecule (i.e., omega-3 or *n*-3 with the first double bond at carbon 3, omega-6 or *n*-6 with the first double bond at carbon 6). Humans lack enzymes to place double bonds in the *n*-3 and *n*-6 positions. These fatty acids are considered essential and are obtained from the diet. The primary *n*-3 polyunsaturated fatty acids (PUFA) in the human diet are alpha-linolenic acid (18 carbons, 3 double bonds), eicosapentaenoic acid or EPA (20 carbons, 5 double bonds), and docosahexaenoic acid or DHA (22 carbons, 6 double bonds). Fish oil represents an important source of DHA and EPA for humans. The primary *n*-6 PUFA in the human diet are linoleic acid (18 carbons, 2 double bonds) and arachidonic acid (20 carbons, 4 double bonds), found in high concentrations in vegetable oils (i.e., soybean, corn, safflower oils). Both *n*-3 PUFA and *n*-6 PUFA are important modulators of inflammation and other immune functions. The modulatory effects of *n*-3 PUFA are the subject of this review.

The immune effects of *n*-3 PUFA have been described as anti-inflammatory, immunomodulatory, and antioxidative [1,2]. These effects contrast with statements regarding *n*-6 PUFA as promoting inflammation and suppressing cell-mediated immunity [1,2]. Immunomodulatory is defined as a substance that affects the functioning of the immune system. The substance may stimulate or suppress immune functioning. Modulation is a non-committal term (i.e., does not express a definite opinion or course of action) and confusing. Descriptions of the immune effects of fatty acids should be concise and consistent. The use of anti-inflammatory and immunomodulatory are also inconsistent since inflammation is a major component of immune functioning. Anti-inflammatory itself indicates a degree of immune suppression. In addition, *n*-3 PUFA are highly susceptible to oxidation due to multiple double bonds. Oxidation of lipids may induce cell damage and stimulate the inflammatory response. Thus, it is unclear whether *n*-3 PUFA are antioxidative or pro-oxidative. The goal of this review is to precisely define the effects of *n*-3 PUFA upon immune functions and oxidative lipid damage. We discuss the effects of *n*-3 PUFA upon both the inflammatory response and immune cell functions, effects upon outcomes using animal models of infection, the relationship between *n*-3 PUFA and resolution molecules, and the effects of *n*-3 PUFA upon lipid peroxidation with and without supplemental vitamin E. It is important to clearly define the effects of *n*-3 PUFA upon immune functioning since the use of an immune stimulant or suppressant has significantly different risks/benefits in individual patient groups.

This narrative review summarizes the evidence for the effects of *n*-3 PUFA upon immune functions, production of resolution molecules, and lipid peroxidation with and without vitamin E. Medline and Embase databases (inception through December 2020) and Google were searched using the terms *n*-3 polyunsaturated fatty acids, fish oil, docosahexaenoic acid, and eicosapentaenoic acid versus immune function, inflammation, infection, resolution molecules, specialized resolution mediators, and lipid peroxidation. The search was limited to English language articles. Studies were included if they were original controlled studies; compared a fish oil supplement or *n*-3 PUFA to a control (i.e., another oil or placebo); reported on one of the endpoints of interest (i.e., immune function, infection, production of resolution molecules, lipid peroxidation); and if reporting immune functions, the results indicated suppression, no effect, or enhancement of immune functions. Adult human, animal, and cellular studies were included. Studies were excluded if the endpoints of interest were not a predetermined outcome of the study and the study was on pediatric patients (<18 years of age). Abstracts, case studies, commentaries, and editorials were excluded. Review articles were retrieved and hand searched to identify additional articles of relevance. A total of 340 articles were retrieved and reviewed. Of the reviewed articles, 133 were included in this review.

## 2. Mechanism of Action of Fish Oil upon Immune Functions

The immune system represents a complex biological system composed of fixed and circulating cells which function in support of innate (nonspecific) and adaptive (acquired, specific) immunity [3,4,5]. These systems protect humans from invading organisms, eliminate abnormal cells (i.e., cancer), and help restore homeostasis following tissue injury. In simplistic terms the immune system can be thought of as composed of three major components: inflammation, cell-mediated immunity, and humoral immunity [3,4,5]. Although inflammation represents an important immune function, many other immune functions are not specific to inflammation (i.e., antigen processing, immunologic memory, immune maturation, cellular proliferation, phagocytosis, suppressor cell functions, helper cell functions, apoptosis, and natural killer cell actions). Thus, when discussing the effects of substances upon immune functions, it is important to be specific.

The biologic effects of docosahexaenoic acid (DHA) and eicosapentaenoic acid (EPA), the primary fatty acids of fish oil, upon inflammation are predominantly stated as anti-inflammatory [6,7,8]. However, the use of the term “anti” may not be the best description of these effects. “Anti” means opposed to. Most effects of *n*-3 PUFA on inflammation result from suppression rather than opposition of inflammation. *n*-3 PUFA suppress the production of transcription factors and cytokines involved in inflammation (discussed below) and produce less-inflammatory eicosanoids [6,7,8]. They do not produce mediators which oppose the actions of inflammatory cytokines. Examples of the anti-inflammatory effects of DHA and/or EPA include suppression of the production of numerous inflammatory mediators that include leukotriene-B4 (LTB4), prostaglandin E2 (PgE2), interleukin-1β (IL-1β), interleukin-2 (IL-2), interleukin-6 (IL-6), interleukin-8 (IL-8), tumor necrosis factor-α (TNF), and reactive oxygen species [6,7,8].

*n*-3 PUFA are also reported to suppress cellular immune responses (Table 1) that include leukocyte chemotaxis, adhesion, proliferation, transmigration, phagocytosis, activation, antigen processing, T-lymphocyte functions, and production of natural killer cells [6,7,8,9,10,11,12,13,14,15,16,17,18,19,20,21,22,23,24,25,26]. Thus, immunosuppressive rather than immunomodulatory is a more accurate scientific term to describe the effects of DHA and EPA. It is these specific inflammatory and cellular immune suppressive effects that form the basis for use of *n*-3 PUFA in the treatment of a variety of inflammatory diseases including rheumatoid arthritis and inflammatory bowel disease [6,7,8,9].

At doses that are administered clinically during nutrition support, DHA and EPA primarily suppress immune functioning [6,7,8]. There is little data to indicate that DHA and EPA directly stimulate immune functions. The literature is prolific with studies documenting the immune suppressive effects of *n*-3 PUFA. We include a sampling of this evidence (Table 1) in discussing the predominant actions of fish oil upon immune functions below [10,11,12,13,14,15,16,17,18,19,20,21,22,23,24,25,26,27,28,29].

A number of investigators have examined the effects of supplemental *n*-3 PUFA upon human leukocytes (Table 1). We first discuss studies in which the *n*-3 PUFA supplement was administered in vivo and then various cells were harvested to access effects upon immune functions. Caughey et al. [10] administered flaxseed oil (high in alpha-linolenic acid, an *n*-3 PUFA) and sunflower oil (high in linoleic acid, an *n*-6 PUFA) to normal volunteers. Following 4 weeks on these oils, both groups received fish oil supplementation (MaxEPA, Scherer Holdings, Troy, MI, USA containing 1.6 g EPA + 1.1 g DHA daily) in addition to the other oils. Flaxseed oil inhibited lipopolysaccharide (LPS)-stimulated monocyte production of TNFα, IL-1β, prostaglandin E2 (PGE2), and thromboxane B2 (TxB2) by approximately 30%. The sunflower oil had little effect on cytokine production. The addition of fish oil decreased cytokine production in both groups. TNFα and IL-1β were decreased approximately 80% while PGE2 and TxB2 were decreased approximately 50%. Endres et al. [11] administered fish oil (MaxEPA, Scherer Holdings, Troy, Michigan, containing 2.8 g EPA + 1.9 g DHA daily) to normal individuals for 6 weeks. Peripheral blood mononuclear cells were harvested. Fish oil supplementation impaired mononuclear cell production of IL-1 and TNFα (stimulated with endotoxin), PgE2 (stimulated with *Staphylococcus epidermidis*), and neutrophil chemotaxis (to LTB4). Lee et al. [12] administered fish oil (MaxEPA, Scherer Holdings, Troy, Michigan, containing 3.2 g EPA + 2.2 g DHA daily) to normal volunteers for 6 weeks. Leukocytes were harvested from the individuals and stimulated with an ionophore. Fish oil suppressed the generation of LTB4 and 5-HETE (5 lipoxygenase product) in both neutrophils and monocytes. Fish oil also impaired neutrophil functioning assessed by endothelial adherence and chemotaxis. James et al. [13] reported that dietary supplementation with different fish oil products (MaxEPA, Scherer Holdings, Troy, MI, USA; Pro-Mega, Parke Davis, Detroit, MI, USA; Pharmacaps, Elizabeth, NJ, USA) containing 1.6–3.5 g EPA + 1.1–2.3 g DHA daily suppressed both TNFα and IL-1β in mononuclear cells in humans. Fisher et al. [14] administered fish oil (cod liver oil containing 3.6 g EPA + 2.4 g DHA daily) for 6 weeks to human volunteers. Monocytes/macrophages were harvested before and after the fish oil supplementation. Fish oil supplementation significantly suppressed stimulated leukocyte production of superoxide and chemiluminescence (a measure of superoxide and other free radicals). These results indicate that fish oils may suppress the monocyte oxygen-dependent respiratory burst, a manifestation of cellular activation.

Wang et al. [21] reported that *n*-3 PUFA found in a linseed oil-based diet (37.5% α-linolenic acid) and fish oil-based diet (Menhaden oil with 14.7% EPA + DHA; 3.4% α-linolenic acid) suppressed lymphocyte proliferation (spleen and thymus, hens) in response to mitogens. Yessoufou et al. [22] reported that an EPA/DHA diet (EPAX oil, Polaris, Quimper, France, *n*-3 PUFA content 24.6 mg/g) compared to a standard diet (*n*-3 PUFA content 0.8 mg/g) in mice impaired T regulatory (Treg) cell suppressive and migratory functions. Treg cells are important for immune tolerance and cancer cell growth. The investigators reported that EPA/DHA downregulated mRNA for chemokine receptors (CCR-4, CXCR-4), curtailed Extracellular Receptor Kinase (ERK) 1/2 and Akt (also known as protein kinase B) phosphorylation, down regulated Smad7 levels (proteins involved in regulation of cell development and growth), inhibited expression of L-selectin mRNA, and upregulated Forkhead Box Protein P3 (Foxp3) mRNA levels and histone deacetylase levels in Treg cells. These molecular changes decrease Treg function.

Similar immune suppressive effects have been demonstrated using fish oil intravenous lipid emulsions. Mayer et al. [23] administered soybean oil (350 mL 10% lipid emulsion) and fish oil (Omegaven, Fresenius Kabi, Bad Homburg, Germany, 350 mL 10% emulsion) as part of total parenteral nutrition to patients with sepsis. The fish oil lipid emulsion suppressed production of IL-1β, IL-6, IL-8, and TNFα compared with the soybean lipid. Furukawa et al. [24] evaluated the effects of fat-free total parenteral nutrition (TPN), soybean lipid (20% of calories) based TPN, and soybean lipid (20% of calories) plus EPA (unspecified preparation, 1.8 g/d) TPN upon the stress response to surgery using IL-6. Compared to no lipid, soybean oil lipid amplified while soybean plus EPA reduced the IL-6 response to surgery. Mayer et al. [25] harvested monocytes from individuals infused with soybean lipid emulsion (350 mL 10% emulsion over 12 h) or fish oil lipid emulsion (Omegaven, Fresenius Kabi, Bad Hamburg, Germany, 350 mL 10% emulsion over 12 h). The fish oil emulsion reduced monocyte pro-inflammatory cytokine generation (TNFα, IL-8) provoked by lipopolysaccharide. Fish oil intravenous lipid emulsion also suppressed monocyte adherence and migration.

In vitro studies of human immune cells also indicate suppression of cellular activities by *n*-3 PUFA. Soyland et al. [15] reported that the purified fatty acids DHA (50–275 µM), EPA (50–275 µM) and arachidonic acid (50–275 µM) suppressed T-lymphocyte proliferation (human, in vitro) stimulated by mitogens and antigens. Zapata-Gonzalez et al. [16] reported that DHA (purified fatty acid, Sigma-Aldrich, St. Louis, MO, USA, 50 µM) inhibited Macrophage Inflammatory Protein 3β (MIP-3β) induced chemotaxis in human monocyte-derived dendritic cells while MIP-1α induced chemotaxis was enhanced. DHA and/or EPA also decreased the production of IL-12, IL-10, and IL-6 (compared to linoleic acid and oleic acid) in dendritic cells. DHA and EPA inhibited lymphoproliferation compared to linoleic acid and oleic acid. Interestingly, DHA downregulated expression of Cluster of Differentiation (CD) 1a, which is involved in the activation of cytolytic T cells (important for resistance to infection). Ferrante et al. [17] reported that arachidonic acid, EPA, and DHA (from Sigma-Aldrich, St. Louis, MO, USA, at doses of 4–10 µg/mL) similarly inhibited human neutrophil migration (random and chemotactic from formyl-methionyl-leucyl-phenylalanine peptide or FMLP). Alpha-linolenic acid, linoleic acid, and oleic acid were without effect. Tull et al. [18] reported that EPA (purified fatty acid, Sigma-Aldrich, St. Louis, MO, USA, 0.05–5 µM) inhibited TNF-stimulated human neutrophil transmigration in endothelial cells. The effect of EPA was reversed with arachidonic acid. Oh and colleagues [19] reported that the anti-inflammatory effects of DHA (purified fatty acid, Cayman Chemical, Ann Arbor, MI, USA 100 µM) upon TNFα, IL-6, and Monocyte Chemoattractant Protein (MCP) 1 in murine macrophages and adipocytes were mediated through the G-protein coupled receptor (GPR) 120, an omega-3 fatty acid receptor. Weatherill et al. [20] demonstrated that DHA (purified fatty acid, Sigma-Aldrich, St. Louis, MO, USA, 5–20 µM) reduced up-regulation of co-stimulatory molecules (CD86, CD40, CD80), the Major Histocompatibility Complex II (MHCII), IL-12p70, and IL-6 in murine bone marrow-derived dendritic cells and implicated the effects via modulation of Toll like receptor-4 (TLR4). DHA was also reported to decrease T-cell activation. Calder et al. [26] demonstrated that fish oil (Omegaven, Fresenius Kabi, Bad Homburg, Germany, 10% emulsion; 36% EPA + DHA) and soybean oil (10% emulsion; 1.6% EPA + DHA) intravenous lipid emulsions both suppressed human lymphocyte proliferation. However, fish oil was more potent than soybean oil.

A thorough search of the literature failed to find many studies that demonstrate an immune-stimulating effect from fish oils. A limited number of studies report enhanced B cell functions. Gurzell et al. [27] evaluated the effects of a standard chow diet (7% fat by weight; based on soybean oil) and a fish oil diet (1% corn oil plus 6% fish oil from MEG-3, ocean nutrition, Newark, California; containing 54% DHA and 20% EPA) upon B lymphocytes from colitis prone SMAD 3 knockout mice. The fish oil diet enhanced B cell functions compared to the chow diet. B cells from the mice fed with fish oil and stimulated with lipopolysaccharide demonstrated enhanced secretion of IL-6 and TNF-α (proinflammatory cytokines) and increased expression of the B cell activation marker CD40 compared to chow controls. There was no difference in ex vivo phagocytosis. Plasma from fish oil fed mice had higher levels of IL-5, IL-13, and IL-9 (Th2-type cytokines) and cecal IgA. Rockett et al. [28] evaluated B cell functions taken from C57BL/6 mice fed a soybean oil-based diet (50 g/kg) or fish oil-based diet (menhaden oil, 50 g/kg). Fish oil enhanced B-cell activation measured with CD69 and cytokine secretion (IL-6, TNFα, IFN-γ). However, fish oil suppressed B-cell stimulation of IL-2 secretion from CD4+ T cells. The effects of *n*-3 PUFA on B cell functions may relate to their effects upon lipid raft structures [27,29]. Interestingly, *n*-3 PUFA enhancement of B cell functions contrasts with their suppressive effects upon T cell functions. Further research into the contrasting effects of *n*-3 PUFA on lymphocyte functions is required so as to better understand their impact upon overall immune functions.

It is important to note that many of the studies discussed above were performed by obtaining blood samples from individuals and patients treated with *n*-3 PUFA. Mediators and cell responses were measured in the blood samples or from harvested cells (usually following ex vivo stimulation to activate the cells). Although these studies help in our understanding of the mechanisms of action of lipids, it remains uncertain if similar responses occur in vivo. However, these are the types of studies that have been used to describe the biologic effects of fatty acids.

Suppression of immune cell functions may lead to increased dissemination of infection. A number of studies using animal models of infection are consistent with such immune suppression (Table 2). Schwerbrock et al. [30] reported that fish oil (Research Diets, New Brunswick, New Jersey) fed mice had impaired resistance to influenza infection. The fish oil group had lower natural killer (NK) cells, neutrophils, and inflammation in the lung and higher mortality compared to the control group. Cruz-Chamorro et al. [31] administered diets based upon olive oil, fish oil (unspecified fish oil), and hydrogenated coconut oil (20% lipid diets) or a low fat diet (2.5%) to mice. Mice were treated with cyclophosphamide to induce an immunosuppressed state or saline (control) and infected with *Listeria monocytogenes*. Splenocyte proliferation was suppressed by fish oil in response to conconavalin A and lipopolysaccharide compared to the other diets in normal and immunosuppressed groups. The fish oil group also had higher levels of bacteria in the spleen and liver compared with the other diets. Mortality was highest with the fish oil diet and lowest with the olive oil diet. The investigators concluded that fish oil was responsible for an immunosuppressed state leading to diminished host resistance to infection and exacerbated immunosuppression from cyclophosphamide. Irons et al. [32] reported that fish oil supplementation (unspecified fish oil) to the diets of mice (compared with a diet high in monounsaturated fatty acids) impaired host resistance to infection with *Listeria monocytogenes* and led to higher mortality. Bonilla et al. [33] studied fat-1 mice with elevated levels of endogenous *n*-3 polyunsaturated fatty acids and reported increased susceptibility to *Mycobacterium tuberculosis* infection and diminished macrophage production of TNFα, IL-6, and IL-1β. In an in vitro study, Bonilla et al. [34] also reported that DHA (purified fatty acid) impaired murine macrophage activation by *Mycobacterium tuberculosis*. DHA diminished macrophage pro-inflammatory cytokine production (TNFα, IL-6, MCP-1) and expression of costimulatory molecules (CD40, CD86), impaired oxidative metabolism (production of reactive oxygen species), and impaired bacterial killing. Interestingly, Zapata-Gonzalez et al. [16] speculate that the high incidence of tuberculosis in Eskimo populations may result from fish oil-induced alterations in antigen presenting cells. Woodworth et al. [35] placed SMAD 3 knockout mice on diets based on corn oil, safflower oil, or corn oil with fish oil (Ocean Nutrition, Newark, California, 0.75%–6.0%). Following 8 weeks on the diets, mice were infected with *Helicobacter hepaticus* (via gavage), which normally causes mild colitis. Fish oil supplementation (2.25%–6.0%) exacerbated colitis, colonic dysplasia and led to the development of adenocarcinoma. These consequences may have been mediated through reduced CD8 T lymphocytes, diminished CD69 expression on CD4 and CD8 T cells, and altered FoxP3 and L-selectin expression on Treg cells. Peck et al. [36] reported higher mortality in burned mice infected with *Pseudomonas aeruginosa* receiving fish oil supplemented diets (MaxEPA, Scherer Corporation, Troy, MI, USA) compared to mice receiving safflower oil supplemented diets or control diets. Chang et al. [37] reported higher mortality in mice infected with oral *Salmonella typhimurium* that received fish oil rich diets (menhaden oil; ICN Biomedical, Costa Mesa, CA, USA) compared to diets rich in corn oil or coconut oil, or a chow diet.

Not all studies demonstrate poorer outcomes from infections with fish oil-enriched diets (Table 2). Bjornsson et al. [38] reported increased survival in mice receiving fish oil-enriched diets (Lysi h.f., Grandavegi, Reykjavik, Iceland) who were infected intramuscularly with *Klebsiella pneumoniae* compared to animals receiving diets enriched with olive oil or standard chow diets. Blok et al. [39] reported improved survival with fish oil supplementation (Mepatrin, Sanofi, Maasslius, Netherlands; Epax oil, Polaris, Quimper, France) in mice infected with *Klebsiella pneumoniae* and *Plasmodium berghei* (causes cerebral malaria) compared to corn oil or palm oil supplemented diets or a chow diet. Interestingly, lipopolysaccharide-induced ex vivo production of IL-1α and TNFα by peritoneal cells was enhanced in the fish oil group.

Other studies have shown no effect of *n*-3 PUFA supplementation on outcomes (Table 2). Clouva-Molyvdas et al. [40] evaluated the effect of supplementation of mouse diets (2–3 weeks) with different lipids (coconut oil; oleic acid; safflower oil; fish oil using MaxEPA from Scherer Corporation, Troy, Michigan; control chow diet with 12% corn oil) upon survival following peritoneal infection with *Pseudomonas aeruginosa* and *Salmonella typhimurium*. There were no significant differences in outcome between diet groups. Barton et al. [41] evaluated a fish oil supplemented diet (Menhaden oil) compared with a safflower oil diet (high in *n*-6 PUFA) in a rat model of abdominal abscess (with *Staphylococcus aureus* and *Bacteroides fragilus*). Although mortality was slightly lower in the fish oil group, the difference was not significant (*p* = 0.4). Fish oil was shown to decrease Kupffer cell production of prostaglandin E2.

In their excellent 2002 review of *n*-3 fatty acids and infectious disease resistance, Anderson and Fritsche [42] reviewed studies of survival in animals infected with various pathogens. Seven studies of Gram negative bacterial infections in mice/rats were reported. Fish oil supplementation increased survival in three studies, decreased survival in two studies, and had no effect on survival in two studies. Five studies reported survival following fish oil supplementation in animals infected with Gram positive bacteria. Two studies reported decreased survival, two studies reported no effect on survival, and one study reported improved survival. Two studies reported decreased resistance to infection with *Mycobacterium tuberculosis*. In regard to viral infections, one study reported increased survival (murine retrovirus), two studies reported delayed clearance with influenza A infection, and one study reported no effect upon survival for cytomegalovirus. Two studies of Plasmodium infection reported improved survival with fish oil. The authors concluded that the data suggest that *n*-3 PUFA can both improve and impair host resistance to infection. The molecular mechanisms that result in these different effects upon infection remains unclear but are worthy of future study.

A few studies have examined the effect of *n*-3 PUFA supplementation upon cancer in animal models. Destruction of cancer cells is dependent upon the endogenous immune system in these models. Impaired immunity would result in increased cancer development and dissemination. Xia et al. [43] demonstrated that fish oil supplementation (fish oil, Bioserv F5424, Flemington, NJ, USA) increased growth of subcutaneously implanted melanoma cells in mice. Woodworth et al. [35] found that fish oil supplementation (fish oil, Ocean Nutrition, Newark, CA, USA) in mice infected with *Helicobacter hepaticus* led to the development of colonic adenocarcinoma and significantly increased mortality compared with control and corn oil-based diets (18% vs. 0%). Mannini et al. [44] compared fish oil supplemented (unspecified fish oil) to maize oil supplemented (high in linoleic acid) diets in mice transplanted with T-lymphoma cells. Fish oil supplementation increased metastasis, cancer organ infiltration, and cachexia. In support of an immune suppressive effect, Grimm et al. [45] demonstrated that fish oil supplementation (fish oil emulsion, unspecified source) prolonged graft survival in an animal heart transplant model (impaired rejection). Interestingly, the immunosuppressive effects of fish oil were similar to those of soybean oil in this model.

It is hypothesized that the hypo-responsive immune environment elicited by fish oil supplementation may play an adverse role in some infections. In contrast, *n*-3 PUFA may reduce inflammation and tissue damage and improve survival with other infections. Importantly, *n*-3 PUFA may impair immunity, which in turn may impair host resistance to infection and cancer and lead to increased mortality. It must be noted that the outcome data cited above was obtained using animal models. Extrapolation of results to the clinical arena should be done with caution since results in animal models may not apply to human disease. Human studies of infection and cancer using *n*-3 PUFA and other lipids are needed to definitively determine their risks vs. benefits.

Investigation of the molecular mechanisms for the immune effects of *n*-3 PUFA supports immune suppression as the primary effect of these fatty acids. These mechanisms include alterations in membrane fatty acid content and lipid rafts [8,27,28], induction of myeloid-derived suppressor cells which impair CD8 T-cell activation and proliferation [43], inhibition of the activation of the pro-inflammatory transcription factor nuclear factor kappa B [8], activation of the peroxisome proliferator-activated receptor gamma [8,16,45,46], modulation of Toll-like receptors [20,47], production of resolvins and protectins [8], and activation of G-protein coupled receptor 120 [19].

Based on the available evidence, the Dietary Reference Intakes (DRIs) from the Institute of Medicine [48] have labeled *n*-3 PUFA with a hazard identification related to immune function. As stated in the DRIs, “All of the single treatment studies comparing individuals fed *n*-3 polyunsaturated fatty acids before and after supplementation showed immunosuppressive effects.”

The suppressive effects of *n*-3 PUFA upon inflammation and immune functions are the basis for the use of fish oils in the treatment of a variety of inflammatory diseases such as rheumatoid arthritis, psoriasis, inflammatory bowel disease, dementia, cachexia, and systemic lupus erythematosus [6,7,9,49,50]. The studies discussed in this article do not imply that the use of fish oils is detrimental to patient care. Whereas the anti-inflammatory and immune-suppressive properties of *n*-3 PUFA may be beneficial for some acute and chronic inflammatory illnesses, these same anti-inflammatory and immune suppressive properties may be detrimental for the response to an infection when an intact immune response is needed to eradicate an invading pathogen. The potential immunosuppressive effects of *n*-3 PUFA may also be deleterious in patients with underlying immunocompromised states.

## 3. Role of *n*-3 PUFA Supplementation upon Levels of Resolution Molecules

*n*-3 PUFA serve as substrates for a family of specialized pro-resolution lipid mediators (SPMs) such as the resolvins, protectins, and maresins [51]. *n*-3 PUFA (i.e., DHA and EPA) derived SPMs are similar in function to *n*-6 PUFA (i.e., arachidonic acid) derived SPMs such as the lipoxins [51]. Thus, both *n*-6 PUFA and *n*-3 PUFA serve as substrates for SPMs.

Normally, the acute inflammatory response is beneficial and protective following injury and infection. The acute inflammatory response is usually self-limited and resolves on its own. Failure to resolve may lead to chronic inflammation that characterizes many inflammatory diseases such as asthma, inflammatory bowel disease, and rheumatoid arthritis. Resolution of inflammation was once thought to be passive and result from loss of the inflammatory stimulus. Today, resolution of inflammation is believed to largely result from an active process initiated through the production of SPMs. These pro-resolving mediators include the lipoxins, resolvins, protectins, and maresins [52,53]. SPMs are hypothesized to contribute to the immune effects of *n*-3 and *n*-6 PUFAs. SPMs are the effectors of inflammatory resolution, with most of their cellular effects resulting from suppression of immune (inflammatory) functions. However, these fatty acid-derived mediators do not act alone but are part of a larger resolution program that includes other mediators such as annexin A1 protein, several cytokines (i.e., TGFβ, IL-10), microRNAs, carbon monoxide, and inhibitors of cyclin-dependent kinases [53]. SPMs modulate inflammation through specific receptors that include ALX (for lipoxin A4, LXA4; resolvin D1, RvD1), chemokine receptor-1 (for resolvin E1, RvE1), G protein-coupled receptor 32 (GPR32) (for RvD1; resolvin D3, RvD3), and G protein-coupled receptor 18 (GPR18) (for resolvin D2, RvD2) [53]. SPM production is triggered by acute inflammation and possess a variety of biologic effects [52,53] that are specific for each molecule but include suppression of the cellular production of cytokines, adhesion molecules, inflammatory transcription factors such as NFkB, and reactive oxygen species. SPMs also impair cellular functions such as neutrophil recruitment, neutrophil infiltration, neutrophil transmigration and chemotaxis. In addition, the SPMs increase uptake and removal of apoptotic cells by phagocytic cells and increase microbial killing. Thus, SPMs possess both immune suppressive effects upon inflammation and immune-stimulating effects (clearance of dead cells and microbes).

The levels of these resolution molecules within blood and tissues are many orders of magnitude lower than the levels of EPA and DHA. For example, DHA is present in tissues at mg/mL levels while resolving D is present in ng/mL levels (a difference of one million). The resolvins, protectins, and maresins are synthesized from EPA and DHA, located primarily in cell membranes. It is unclear to what degree SPMs contribute to the immunologic effects of *n*-3 PUFA. Clearly, DHA and EPA produce immune effects through pathways that differ from those of SPMs. In this section, we review studies that address the question of whether *n*-3 PUFA supplementation increases SPM levels in both healthy and diseased individuals. Lack of a consistent effect upon SPM production would support a diminished role for SPMs in *n*-3 PUFA cellular effects.

A number of studies have evaluated the effect of *n*-3 PUFA supplementation upon the production of SPMs in both healthy individuals and patients with chronic diseases (Table 3). Markworth et al. [54] administered EPA (Maxomega EPA, Equateq, Breasclete, United Kingdom) or docosapentaenoic acid (DPA; Maxomega DPA, Equateq, Breasclete, United Kingdom) (2 g initial, 1 g/d for 6 additional days) or olive oil (placebo) to 10 healthy female volunteers in a double-blind cross-over study. DPA supplementation increased 13-monohydroxylated DPA, 19,20-dihydroxy DPA, 7,17-dihydroxy DPA, and maresin 1 levels. There was no change in resolvin D1 levels. DPA also increased the *n*-6 PUFA metabolites of PGE2. EPA increased numerous hydroxyl-eicosapentaenoic acid levels (including 18-hydroxyeicosapentaenoic acid; 18-HEPE), but failed to change levels of maresin-1, resolvin D1, or resolvin E1. Levels of various hydroxyl-eicosatetraenoic acids (HETEs derived from arachidonic acid) were decreased. Barden et al. [55] administered fish oil (Omega Daily, Blackmores, Warriewood NSW, Australia; 2.4 g/d EPA + DHA) to 21 healthy volunteers. Following 5 days of treatment there was an increase in plasma DHA, EPA, resolvin E1, 18R/S-HEPE, 17R/S-HDHA (hydroxydocosahexaenoic acid), and 14R/S-HDHA. However, there were no increases in resolvins E2 and E3, 18R-resolvin E3, resolvin D1 and D2, or 17R-resolvin D1. Addition of aspirin for 2 additional days failed to have effects on SPM levels. Colas et al. [56] studied 10 healthy volunteers 4 h following 0.7 g EPA + DHA (Sundown Naturals, Bohemia, NY, USA; with 81 mg aspirin). There was an increase in plasma levels of resolvins D1 and D2, 17-epi-protectin D1, resolvin E2, and resolvin E3. There were decreases in plasma levels of 17-epi-resolvin D1, resolvin D3, resolvin D6, 17-epi-resolvin D3, and resolvin E1. There was no change in levels of resolvin D5. Norris et al. [57] administered *n*-3 PUFA supplements (Nordic Naturals, Watsonville, California; EPA + DHA 0.9, 1.8, 3.4 g/d) to a small number of healthy individuals (*n* = 6) for 2 and 5 months. An SPM cluster representing resolvin D1, resolvin E1, lipoxin (LX) B4, aspirin triggered lipoxin A4 (AT-LXA4), 18-hydroxy-eicosapentaenoic acid (18-HEPE), and 17-hydroxy-docosahexaenoic acid (17-HDHA) were measured following the supplementation period and following low dose intravenous lipopolysaccharide (LPS). In study A, 6 individuals were randomized to receive soybean oil (placebo) or EPA + DHA (as triglycerides, 900 mg/d or 1800 mg/d). In study B, 3 individuals were randomized to 3.4 g/d DHA + EPA (as ethyl esters) or olive oil as a placebo in a cross-over study. Study A used an *n*-3 PUFA preparation based on triglycerides while study B used fatty acids based on their ethyl esters. Despite the small number of patients, the results indicate that *n*-3 PUFA supplementation increased levels of the cluster SPMs prior to administration of LPS. The SPMs in the cluster were elevated at baseline compared to the placebo in study groups A (950 vs. 250 pg/mL) and B (100 vs. 30 pg/mL). Levels of the SPMs, in study A, decreased over the first few hours after LPS and then returned to baseline values at 24 h, where they remained at or close to baseline through the study (120 h). Interestingly, the SPM response was different in study B, where they increased over the first 8 h after LPS and then returned toward baseline values at 24 h and remained at or close to baseline through 168 h. The differences in response to LPS may reflect upon the different *n*-3 PUFA preparations used, the different durations of treatment, and use of serum (study A) or plasma (study B). The decrease in SPM levels following LPS in study A is consistent with impaired SPM synthesis following an acute inflammatory state.

SPMs have also been evaluated in patients with chronic inflammatory conditions (Table 3). Ramirez et al. [58] studied the effect of *n*-3 PUFA supplementation (ProOmega, Nordic Naturals, Watsonville, CA, USA) upon SPMs in patients with peripheral artery disease. Patients were randomized to fish oil 4.4 g/d (*n* = 11; containing 2.2 g EPA + DHA) or soybean oil (placebo, *n* = 13) for 3 months. Fish oil significantly increased plasma levels of EPA and DHA. Overall, fish oil significantly increased levels of HEPE (5-, 11-, 12-, 15-, 18-), lipoxin A5, resolvin E1, resolvin E3, and HDHA (4-, 7-, 13-, 14-, 17-, 21-). However, there was no increase in levels of resolvin E2, protectin D1, maresin 1 or 2, and resolvins D1, D2, D3, D4, D5, 17R-D1, or 17R-D3. These results suggest an impairment in the metabolism of 17-HDHA to resolvins in patients with peripheral vascular disease. Interestingly, the increase in HEPEs and resolvins E1 and E3 with fish oil was extremely heterogeneous. Although 18-HEPE showed consistent increases with fish oil, the change in resolvin E levels ranged from a small decrease or no effect to an increase in levels. For example, resolvin E1 decreased in 1 patient, was unchanged in 1 patient, and demonstrated a small or larger increase in the remaining patients. In the placebo group, resolvin E1 decreased in 2 patients and increased in the remainder. The 2 patients with the largest increases in resolvin E1 were in the placebo group (the increase being 3 fold greater than any patient in the fish oil group). Similar heterogeneous responses were found for resolvin E3.

Keelan et al. [59] administered fish oil (unspecified source; 4 g/d containing 3.7 g of *n*-3 PUFA) or olive oil (4 g/d) to 51 healthy pregnant women beginning at 20 weeks gestation (randomized study). Maternal blood and placenta were obtained following delivery. DHA and EPA increased in maternal erythrocytes following fish oil supplementation. In the placenta, DHA also increased (2 fold) in the fish oil group. Levels of EPA, ALA, and DPA were not increased in the placenta following fish oil supplementation. The SPM precursors, 18-HEPE and 17-HDHA, were increased in placenta following fish oil. However, there was no increase in resolvin D1, 17R-resolvin D1, resolvin D2, or protectin D1 levels in the placenta in either group. Interestingly, TNFα gene expression in placenta increased 14 fold in the fish oil group while levels of IL-1β, IL-6, and IL-10 were similar between groups. Thus, fish oil supplementation increased levels of resolvin and protectin precursors in placenta but failed to increase levels of the resolvins and protectins themselves. The molecular basis for the failure to convert DHA and EPA to resolvins and protectins is unclear. Placenta normally contains significant levels of both 5-LOX (lipoxygenase) and 15-LOX enzymes [59].

In a small open label study of 6 patients with coronary artery disease receiving fish oil supplements (*n* = 3; 3.36 g/d DHA + EPA) or no supplement (*n* = 3) for 1 year, Elajami et al. [60] reported that fish oil supplementation (Lovaza, GlaxoSmithKline, Research Triangle Park, North Carolina) increased levels of resolvins D6 and E2, aspirin-triggered resolvin D3, aspirin-triggered protectin D1, and aspirin-triggered lipoxin B4. However, there were lower levels of aspirin-triggered resolvin D1, resolvin E3, and lipoxin B4. Overall, SPMs were similar between groups (593 pg/mL fish oil vs. 605 pg/mL placebo) with resolvins and protectins higher in the fish oil supplemented group and lipoxins higher in the placebo group. Barden et al. [61] evaluated the effect of fish oil supplementation (Omega Daily, Blackmores, Warriewood NSW, Australia; 4 g/d fish oil containing 2.4 g/d EPA + DHA for 3 weeks) in patients with metabolic syndrome (*n* = 22) and matched controls (*n* = 21). Following 3 weeks of fish oil, aspirin was added to the fish oil for another week. Fish oil increased plasma EPA and DHA levels and 18-HEPE levels in both groups, although the increase in 18-HEPE was lower in the metabolic syndrome patients. 17-HDHA and 14-HDHA increased in the control group. However, there was no change in 17-HDHA and 14-HDHA levels in the metabolic syndrome patients. Resolvins E1-E3 increased similarly in both groups following fish oil. However, there was no increase or a slight decrease in resolvins D1-D2, 17R-resolvin D1, protectin D1, and maresin-1 in both groups. Addition of aspirin was without effect. This study demonstrates impaired metabolism of EPA and DHA to 18-HEPE, 17-HDHA, or 14-HDHA in patients with metabolic syndrome. It also demonstrates the ineffectiveness of fish oil for increasing production of resolvins D1-D2, protectin D1, and maresin-1 in healthy and metabolic syndrome patients.

Fiala et al. [62] evaluated the effect of *n*-3 PUFA supplementation (Smartfish, Oslo, Norway; 2 g/d EPA + DHA over 4-17 months) upon resolvin D1 levels in macrophages from patients with cognitive impairment. Resolvin D1 levels over time were extremely variable. Levels increased in six patients, decreased in four patients, and fluctuated up and down in four patients. There appeared to be no consistent change following the *n*-3 PUFA supplementation. Mas et al. [63] studied patients with chronic kidney disease. Twenty patients received a fish oil supplement (Omacor, Solvay Pharmaceuticals, Pymble NSW, Australia containing 3.5 g/d EPA + DPA + DHA) while 15 received a placebo (olive oil) for 4 weeks. The fish oil supplement increased plasma levels of 18-HEPE, 17-HDHA, and resolvin D1. There was no change in levels of 17R-resolvin D1 or resolvin D2. Wang et al. [65] reported decreased mononuclear cell resolvin D1 production from fish oil supplements (EPAX1050TG, Pronova Biocare, Lysaker, Norway; 1.7 g/d DHA, 0.6 g/d EPA) in patients with Alzheimer ’s disease. Fish oil supplementation (*n* = 8) was compared with corn oil supplementation (*n* = 7) over 6 months of treatment. Fish oil increased plasma levels of DHA and EPA. However, there was a decrease in resolvin D1 levels in both groups (greater in corn oil group). In the fish oil group, resolvin D1 increased in two patients, was unchanged in one patient, and decreased in five patients. There were no differences between groups for lipoxin A4.

The previous studies indicate that the change in SPMs following fish oil supplementation is extremely variable (some SPMs increase, others show no change) and suggest that fish oil, DHA and EPA may not be the best substrates for increasing synthesis of SPMs. It is possible that slow or minimal conversion of fish oil supplements to SPMs could be bypassed by the administration of SPM precursors that are further down the metabolic pathway and closer structurally to the SPMs. For example, Souza et al. [64] evaluated the effect of a novel marine oil supplement upon peripheral blood pro-resolving mediator concentrations and immune responses in healthy volunteers. In a double-blind, placebo-controlled, crossover study individuals were administered placebo or 1.5, 3.0, and 4.5 g of the novel supplement (composed of 18-hydroxy-eicosapentaenoic acid and 17-hydroxy-docosahexaenoic acid) [57,66]. Blood was obtained at baseline, 2, 4, 6, and 24 h after the supplement. SPMs (resolvins and protectins) were found to increase after the 3 g dose (peak 2 h, duration 6 h) and 4.5 g dose (peak 2–4 h, duration 24 h) of the supplement. The 1.5 g dose and placebo had no effect on SPMs. The investigators evaluated the effect of the novel supplement upon adhesion molecule expression in monocytes, neutrophils, and platelets as a measure of cellular activation. Monocyte expression of CD11b and CD162 were suppressed by the supplement. Neutrophil expression of CD11b (at 6 h) and CD49d (24 h) were upregulated. At 24 h post supplement, CD11b was suppressed. Expression of CD41 was suppressed on monocytes. Expression of CD63, a platelet activation marker, was suppressed by the supplement. Assessment of adhesion molecule expression following stimulation with platelet activating factor (PAF) indicated that the novel supplement suppressed expression of monocyte CD11b and CD49d while upregulating expression of CD16. The supplement also increased expression of CD49d on neutrophils. Supplementation decreased monocyte-platelet aggregates but increased platelet-neutrophil aggregates following PAF. Blood was incubated with fluorescent labeled bacteria (*E. coli* and *S. aureus*) for assessment of phagocytosis. The supplement increased phagocytosis of both bacteria by neutrophils and monocytes. The overall effect of the supplement is difficult to interpret since cellular activation markers were both upregulated and down regulated. However, the results indicate that a supplement composed of SPM precursors may increase production of resolvins and protectins.

Defects in SPM pathway metabolism and action can impair inflammatory resolution and may lead to chronic inflammation [53,57,67]. There may also be alterations in SPM pathways during acute disease that impair resolution or result in exaggerated and prolonged inflammation. These defects include impaired receptor expression, enzymatic synthesis, intracellular signaling, or deficiencies in substrate supply (i.e., PUFA). Receptor dysfunction may result from genetic polymorphisms in enzymes for SPM receptor synthesis, dysfunctional SPM receptors, and/or decreased SPM receptor expression.

Impaired or dysfunctional pathways for SPMs have been reported in a variety of diseases that include chronic obstructive pulmonary disease (COPD), diabetes mellitus, obesity, rheumatoid arthritis, atherosclerosis, heart failure, inflammatory bowel disease, neurodegenerative diseases, and others [67]. For example, Levy et al. [68] and Planaguma et al. [69] reported decreased biosynthesis of SPMs in patients with asthma. Miyata et al. [70] reported decreased synthesis of protectin D1 (PD1) by eosinophils from patients with severe asthma (compared with healthy subjects), even in the presence of DHA. Croasdell et al. [71] reported decreased levels of resolvin D1 in patients with COPD. Arnardottir et al. [72] reported decreased levels of serum resolvin D3 (4.3 ± 1.4 vs. 17.7 ± 2.3 pg/mL, *p* = 0.008) and resolvin D4 (1.1 ± 0.3 vs. 3.3 ± 0.9 pg/mL, *p* = 0.08) in patients with rheumatoid arthritis compared to healthy controls. Wang et al. [65] reported decreased mononuclear cell resolvin D1 production from patients with Alzheimer’s Disease. Merched et al. [73] report impaired production of SPMs in atherosclerosis. Fredman et al. [74] reported lower 5-LOX derived SPMs, especially resolvin D1, in human vulnerable vs. stable atherosclerotic plaques. Simiele et al. [75] reported a single nucleotide mutation (SNP) in the *FPR2/ALX* gene which impaired expression of the G-protein coupled receptor for lipoxins in a patient with cardiovascular disease. Reina-Couto et al. [76] reported lower levels of lipoxins in patients with higher severities of chronic heart failure compared to those with lower severities of chronic heart failure. Chiurchiu et al. [77] reported decreased plasma resolvin D1 levels (2 fold lower) in patients with congestive heart failure (CHF) vs. controls. The lower plasma resolvin D1 levels were associated with a 5-fold reduction in leukocyte 15-lipoxygenase mRNA, suggesting decreased biosynthesis of resolvin D1. The investigators next evaluated the effects of both resolvin D1 and resolvin D2 upon CD8 and CD4 T-lymphocyte pro-inflammatory cytokine production. Neither resolvin molecule modulated cytokine production in leukocytes from CHF patients, suggesting impaired T-cell responsiveness to these SPMs. Further study found that leukocyte receptors for resolvin D1 (GPR32 and ALX/FPR2) were decreased in patients with CHF. Thus, unresponsiveness to these SPMs may result from impaired synthesis of their receptors.

In patients with impaired SPM metabolism and receptor functions, administration of dietary fatty acids such as DHA and EPA may not be the most efficient method to augment conversion to SPMs. Administration of SPMs or SPM analogs may be more effective. Administration of SPMs to individuals with chronic inflammation makes clinical sense since these patients may have defective SPM synthesis or actions. However, it is unclear if administration of SPMs to previously healthy individuals with acute inflammation would be beneficial. Inflammation resolves spontaneously in most of these individuals and it is possible that too early administration may result in a detrimental curtailment of inflammation.

The status of SPM pathways in acute inflammatory diseases in humans is insufficient at this time to generate conclusions about its functions. The effect of *n*-3 supplementation upon target tissue uptake and conversion to SPMs, as well as receptor and post receptor pathways, needs further study. Dalli et al. [78] measured cytokines, prostaglandins, leukotrienes, and SPMs in 22 medical intensive care unit (ICU) patients with sepsis over 7 days following hospital admission. The investigators compared survivors (*n* = 13) with non-survivors (*n* = 9). Higher levels of pro-inflammatory cytokines (i.e., TNFα, IL-6, IL-8), prostaglandin F2α, leukotriene B4, and SPMs (resolvin D5, resolvin E1, 17-epi-resolvin D1, and 17-epi-protectin D1) correlated with higher mortality. The investigators speculate that failure of resolution (i.e., response to the SPMs) contributed to the mortality. Thus, in this study higher SPM levels failed to improve outcome but may reflect upon defects in action rather than synthesis.

The SPM response to microbial infection is complicated. Some organisms may suppress the production of SPMs (i.e., influenza viruses) [53]. More virulent strains of influenza virus are associated with suppression of lipoxin production and enhanced dissemination. *Influenza H5N1* has been shown to downregulate protectin D1 levels. These individuals may benefit from the administration of specific SPMs. In contrast, other infections appear to generate local SPMs which assist with immune evasion and/or decreased survival (i.e., *Pseudomonas aeruginosa*, *Candida albicans*, *Mycobacterium tuberculosis*) [53]. For example, Bafica et al. [79] studied *Mycobacterium tuberculosis* infection in mice. Wild type mice demonstrated increased production of both LTB4 and LXA4 following infection. Mice deficient in 5-lipoxygenase (involved in the synthesis of leukotrienes, resolvins, and lipoxins) failed to increase production of LXA4 and LTB4. The lipoxygenase deficient mice had lower bacteria in lungs and spleen, decreased pulmonary inflammation, increased pro-inflammatory mediator production (IL-12, IFN-γ, nitric oxide synthase 2), and enhanced survival. Administration of an LXA4 analog to the 5-lipoxygenase deficient mice reversed the benefits of lowered LXA4 manifested by lower IFN-γ and increased bacterial counts in the lung and spleen. These results suggest that SPMs impair resistance to some infections and that inhibition of SPMs represents a strategy for improving outcome from selected infections. On the other hand, Aliberti et al. [80] studied mice (wild type and 5-lipoxygenase deficient) infected with the parasite *Toxoplasma gondii*. *Toxoplasma gondii* induced a marked increase in LXA4 levels in the mice while no increase was seen in lipoxygenase deficient mice. The deficient mice had higher pro-inflammatory mediator levels (IL-12, IFN-γ, TNF), greater tissue damage, lower numbers of brain cysts, and decreased survival compared to the wild type mice. Administration of a LXA4 analog improved survival in the deficient mice. Bannenberg et al. [81] demonstrated that *Toxoplasma gondii* caries 15-lipoxygenase activity (required for synthesis of lipoxins, resolvins, and protectins). *Toxoplasma gondii* is capable of generating LXA4 when supplied with arachidonic acid (which normally originates in tissue cells during infection). Interestingly, these investigators also demonstrated that administration of soybean 15-lipoxygenase (intraperitoneally) promotes synthesis of LXA4 during murine peritonitis [81]. The study suggests that administration of lipoxygenases may also represent a method for augmentation of SPM synthesis. The two studies discussed above illustrate the delicate balance between pathogen, host immune response, levels of SPMs, and survival. In one case (Mycobacterium infection), high LXA4 was associated with decreased survival and in the other case (Toxoplasma infection) high LXA4 was associated with improved survival. Thus, it is important that clinical trials evaluate outcomes from SPM modulations (i.e., administration or inhibition) in specific diseases and infections and that results not be extrapolated from one insult to another.

The biological demand for an initial robust inflammatory response against a microbial insult must be balanced against the need to control an exaggerated inflammatory response (with tissue injury) and prevention of a prolonged response (chronic inflammation). If given too early during the inflammatory response, SPMs may impair microbial clearance and worsen survival. This premise is supported by the study of Sordi et al. [82] using an animal model of pulmonary sepsis (intratracheal *Klebsiella pneumoniae*). LXA4 levels increase early in this infectious model, along with the inflammatory response. Treatment with LXA4 receptor antagonists (antagonizes SPM action) early during sepsis decreased bacterial dissemination and improved survival. Early treatment with LXA4 (increased SPM action) worsened infection and failed to improve survival. In contrast, treatment with LXA4 during late sepsis improved inflammation and survival while LXA4 receptor antagonists had no effect. The study demonstrates a time window in sepsis when anti-inflammatory and pro-resolving therapies may be beneficial or harmful. Much like anti-inflammatory therapies, clinical trials are required to determine the optimal timing and benefit vs. harm of pro-resolving therapies.

It is hypothesized that the control over SPM production rests in the metabolic pathways required for their production and action. Modulation of enzymatic activity for metabolism of *n*-3 PUFA and *n*-6 PUFA substrates to SPMs (by up or down regulation of enzymatic levels) and receptor activity is more likely to determine activity of these pathways than substrate supply in most individuals. This is not to state that substrate is not important. There must be an ample supply to allow for metabolism to SPMs. However, augmenting supply of substrates above levels required for efficient metabolism is unlikely to increase levels. Defects in the metabolic pathways for SPM production are best approached by increasing enzymatic activity or administering the active metabolites of SPMs. Direct administration of specific SPMs can optimize the dose effect and tissue specificity of the response much better than administration of precursors. Future studies will need to explore the best techniques for augmenting SPM production, timing of SPM intervention, and specific diseases where impairment of synthesis might occur (i.e., inflammatory states, chronic disease).

Administration of LXA4 as well as resolvin analogs has been shown to be beneficial in various experimental disease models. It remains unclear which of these SPM classes is most beneficial in various disease states. Production of SPMs may be tissue-specific and the benefits of SPMs in various diseases may be SPM specific. Clinical trials will be required to demonstrate the benefits or harm of various SPMs and SPM precursors in a variety of diseases. SPMs are produced in many, if not all, tissues. Their actions are believed to be local and short lived. It is unclear if levels in the blood accurately reflect upon levels in the tissues. SPMs also appear to be tissue and disease specific. Not all SPMs are produced in each tissue [83]. It is essential that studies evaluate the tissue specificity of SPM actions and tissue-specific SPM production in a variety of human diseases.

It is interesting that some clinicians advocate administration of *n*-3 PUFAs in an attempt to augment production of resolvins/protectins/maresins. However, I am not aware of any advocating administration of *n*-6 PUFA (i.e., in soybean oils) in an attempt to augment synthesis of lipoxins. Although both classes of PUFA suppress many cellular immune functions, studies indicate that *n*-6 PUFA may increase production of inflammatory mediators while *n*-3 PUFA decrease production of inflammatory mediators. Perhaps the choice of substrate should depend upon the underlying level of inflammatory mediators.

The variable levels of different SPMs in different individuals and the heterogeneous effects of *n*-3 PUFA supplementation and disease states may affect interpretation of results from selective measurements of SPMs. Thus, SPM profiling (measuring all the major SPMs) may be important for understanding the overall status and effects of treatments related to disease resolution in various disease states. This approach is similar to measurement of cytokines during various inflammatory insults. One cytokine level may be inadequate for describing the complete response. The choice of sample may also be important. Colas et al. [56] demonstrated that serum had significantly higher SPM levels than found in plasma. The increase is believed to reflect changes that occur with ex vivo clotting of blood from serum samples [56,57]. Mas et al. [84] compared levels of SPMs in serum and plasma. Levels of 18-HEPE and 17-HDHA were higher in plasma than serum while levels of resolvins D1 and D2, and 17R-resolvin D1 were comparable. Thus, studies should be performed using plasma samples of SPMs. The differences between serum and plasma also suggest that differences in levels (due to degradation), as well as activation status of blood cells, may affect SPM measurements. Synthesis of SPMs may be selective to certain tissues. Thus, levels of SPMs should be obtained from relevant tissues during clinical studies, if at all possible. Levels in blood may not reflect levels in liver, lung, or other tissues. The dose of *n*-3 PUFA or SPM, type (form of drug substrate) of *n*-3 PUFA or SPM supplement, type and dose of placebo (i.e., olive oil, soybean oil, no lipid), duration of treatment, underlying disease, acute vs. chronic duration of disease, and effect of the inflammatory status of patients are all variables that affect clinical response and benefits of SPM therapies. Finally, due to the heterogeneous response noted in clinical trials using lipid precursors and SPMs, the sample size should be large enough to account for variability.

In summary, SPM production varies with disease activity and appears diminished in patients with a variety of chronic diseases. Fish oil (*n*-3 PUFA) supplementation fails to produce consistent increases in SPM levels in both healthy individuals and patients with chronic diseases. SPM actions are best augmented using SPM analogs. SPM administration may have both beneficial and detrimental effects depending upon stage of disease (i.e., early vs. late).

## 4. *n*-3 PUFA Supplementation and Lipid Peroxidation

Oxidants are normally produced during the oxidation of carbohydrates, lipids, and amino acids for the production of energy [85]. Thus, oxidation is a normal metabolic process. Oxidants play important roles in cell signaling and killing of pathogens and malignant cells. However, oxidants may also damage structural components of cells such as membranes, proteins, and nucleic acids and oxidants are thought to contribute to numerous diseases [86,87,88]. The cell has evolved a variety of mechanisms to protect itself from excessive oxidant production and to repair oxidant damage. Oxidant stress occurs when oxidant production exceeds the ability of the cell to neutralize oxidant effects [86,88]. The excess oxidants that are produced damage tissues which can result in disease, impaired organ functions, and early death. Reactive oxygen species and lipid peroxidation products are well known to induce inflammation and other immune alterations that include increased cytokine secretion and activation of inflammatory transcription factors [86,87,88]. Lipid peroxidation products have longer biologic half-lives than free radicals and can diffuse from sites of formation to other sites within cells, causing damage to vital metabolic components. Since *n*-3 PUFA are susceptible to oxidative damage which may increase inflammation, we review the effects of *n*-3 PUFA supplementation upon lipid oxidation.

Oxidation of lipids, proteins, or nucleic acids can be assessed using tests for specific oxidation products [89,90,91,92,93]. For example, F3-isoprostanes represent oxidation of EPA [92]. F2-isoprostanes represent oxidation derived from arachidonic acid, and F4-neuroprostanes derive from oxidation of DHA [90,91,92,93]. In addition, the aldehydes 4-hydroxynonenal (HNE) derive from *n*-6 PUFA oxidation while 4-hydroxyhexenal (HHE) derive from *n*-3 PUFA oxidation [93]. There are also specific oxidation products that derive from protein and nucleic acid oxidation [89]. It is common to measure peroxidation of arachidonic acid when assessing oxidative stress because arachidonic acid represents one of the major fatty acids of cell membranes. An increase in arachidonic acid peroxidation products does not indicate that this fatty acid is the cause of the increased oxidation but rather it is an indicator of the presence of oxidative stress. It may well be that some other agent is the cause of increased oxidative stress which is reflected by increased membrane oxidation of arachidonic acid. Some degree of low grade oxidation is normal and may be protective by upregulating anti-oxidant systems and repair pathways that are protective. However, high grade oxidation damages tissues and is detrimental [86,87,88].

Similar to the use of other biochemical tests, it is important that one measure the specific products that represent the appropriate pathways under investigation. If one is interested in *n*-3 PUFA oxidation, one should measure an *n*-3 PUFA peroxidation product. Oxidation products that directly assess oxidative damage to proteins, nucleic acids or lipids are preferable to measuring substances that indirectly assess increased oxidant production (such as vitamin E homologs, and anti-oxidant enzymes). Anti-oxidant substances and enzymes [94,95] may be consumed in the process of neutralizing oxidants, without resulting in damage to tissues. Thus, measuring substances which directly access tissue oxidant damage is preferable over tests that indicate increased oxidant production. Unfortunately, most published studies that evaluate oxidant stress do not measure substances that directly evaluate lipid peroxidation or end organ effects of oxidants or oxidants themselves. Most studies report on levels of various anti-oxidants [94,95]. In addition, one must be careful when administering anti-oxidants not to cover up oxidant damage. For example, administering vitamin E and then using vitamin E levels to access oxidation may result in erroneous conclusions that there is no increase in oxidation. Vitamin E levels may increase in the face of increased oxidative damage.

The study of Miloudi et al. [96] is an example of why measuring the appropriate oxidation products is important for interpreting studies of lipid oxidation. Miloudi investigated oxidation of a variety of parenteral nutrition formulations containing a soybean oil-based lipid emulsion or a fish oil-based lipid emulsion (Omegaven, Fresenius Kabi, Bad Homburg, Germany). Oxidation was evaluated both in vitro and in vivo (guinea pigs infused with the formulations). Oxidation was assessed using hydroperoxides, two aldehydes, and F2α-isoprostanes. *n*-6 PUFA peroxidation was assessed using 4-hydroxynonenal (HNE) and F2α-isoprostanes while *n*-3 PUFA peroxidation was assessed using 4-hydroxyhexenal (HHE). Total PUFA content of the two lipid emulsions were not equivalent; total PUFA content was twice as high in the soybean lipid emulsion formulation compared with the fish oil emulsion formulation (approximately 25 vs. 12 mM). When comparing peroxidation between different lipid emulsions it is very important to administer comparable quantities of fatty acids. Despite higher PUFA administration with the soybean emulsion, total hydroperoxides were comparable between the two lipid emulsions. As expected, HNE levels were higher in the soybean emulsion compared to the fish oil emulsion while HHE levels were higher in the fish oil emulsion compared to the soybean lipid emulsion. However, the sum of HNE and HHE were higher with the fish oil emulsion compared to the soybean emulsion (approximately 15.3 µM vs. 4.1 µM). A fatty acid oxidation index was calculated and indicated that *n*-3 PUFA were more susceptible to oxidation than *n*-6 PUFA. Hepatic levels of F2α-isoprostanes (reflecting peroxidation of *n*-6 PUFA) were slightly (but not significantly) higher in the soybean lipid group. Although hepatic levels of glutathionyl-HNE (GS-HNE) adduct were higher in the soybean lipid group, levels of glutationyl-HHE (GS-HHE) were higher in the fish oil lipid group. The sum of GS-HNE and GS-HHE were similar between lipid emulsion groups. Thus, to obtain an accurate assessment of lipid peroxidation it is essential to measure levels of both *n*-6 PUFA and *n*-3 PUFA peroxidation, as well as total lipid and other oxidative products. Measurement of one lipid peroxidation product is unreliable for making conclusions about total lipid peroxidation.

Double bonds within fatty acid molecules are preferential sites for oxidation. Due to the large number of double bonds in *n*-3 PUFA (EPA has five double bonds, DHA has six double bonds), these molecules are particularly susceptible to lipid peroxidation in lipid emulsions and within cells. Studies indicate that the peroxidizability of unsaturated fatty acids increases with the number of double bonds [97]. Holman reported the relative oxidation rates for unsaturated fatty acids to be 0.025 (1 double bond), 1 (2 double bonds), 2 (3 double bonds), 4 (4 double bonds), 6 (5 double bonds), and 8 (6 double bonds) [97]. Thus, DHA (containing 6 double bonds) has an oxidation rate 320 times higher than oleic acid (1 double bond).

Lipid peroxides result from oxygen free radical damage to cell membranes and may result in cell injury and death. Lipid peroxides are also mutagenic. There is more than ample evidence from basic research studies in cells, animal studies, and human studies to demonstrate increased lipid peroxidation with long chain *n*-3 PUFA. A small sampling of the research is discussed below (Table 4).

Xu and colleagues [98] reported higher lipid peroxidation in a fish oil intravenous lipid emulsion (Omegaven, Fresenius Kabi, Bad Homburg, Germany) compared to an olive soybean intravenous lipid emulsion. Watkins et al. [99] studied the production of oxidants in colonic cells. Oxidant production was enhanced with DHA (purified fatty acid) and lowest with oleic acid (a monounsaturated fatty acid). Watkins found that DHA accumulates in cardiolipin, an important lipid component of the inner mitochondrial membrane. Accumulation of highly unsaturated fatty acids such as DHA in proximity of the mitochondrial electron transport chain, the primary source of oxidants within the body [125], makes DHA particularly susceptible to oxidant damage. These results were confirmed by Ng et al. [100] who demonstrated that DHA (purified fatty acid) increased oxidation and apoptosis in colonocytes compared to linoleic acid or no lipid supplementation.

Fuhrman et al. [101] evaluated serum oxidation in mice supplemented with various fatty acids or oils. DHA (purified fatty acid) and fish oil (unspecified) enhanced serum oxidation compared to oleic acid, olive oil, linoleic acid, and soybean oil. In addition, fish oil decreased serum paraoxonase-1 activity, an enzyme which hydrolyzes oxidized lipids. Kubo et al. [102] evaluated the effect of DHA supplementation (from sardine oil; DHA 0 to 8.7% of energy) and vitamin E supplementation (54–402 mg/kg) upon lipid peroxidation in rats. Results indicated that DHA enhanced susceptibility of the liver and kidney to lipid peroxidation concomitant with higher levels of DHA in the tissues. Vitamin E was unable to protect membranes of the liver and kidney rich in DHA from lipid peroxidation. Oarada et al. [103] administered fish oil (NOF corporation, Tokyo, Japan; 13.5% weight fish oil, 1.5% weight soybean oil), soybean oil (15% weight), or olive oil (15% weight) to mice for 3 weeks. Fish oil supplemented mice had higher levels of TBARS (thiobarbituric acid reactive substances; a measure of lipid peroxidation) in their livers and kidneys compared to soybean oil and olive oil fed mice. Song et al. [52,104] administered a palm oil/soybean oil diet with or without DHA (Bizen Chemical, Okayama, Japan) to rats (lipid 15% weight of diet). DHA intake increased lipid peroxidation (TBARS, hydroperoxides) in plasma, kidney and liver compared to control rats. The investigators concluded that dietary DHA enhances susceptibility of plasma and tissue membranes to lipid peroxidation concomitant with higher levels of *n*-3 PUFA in membrane phospholipids. Lavoie et al. [105] used an animal model (neonatal guinea pig) of pulmonary alveolar development and evaluated the impact of parenteral nutrition with a soybean intravenous lipid emulsion versus a fish oil containing intravenous lipid emulsion (SMOFlipid, Fresenius Kabi, Bad Homburg, Germany; containing soybean oil, medium chain triglycerides, olive oil, fish oil). Increased oxidative stress inhibits alveolar development in this model. The fish oil containing lipid emulsion increased oxidative stress and apoptosis and impaired alveolar development compared with the soybean lipid emulsion. Lengo et al. [106] evaluated oxidation and DNA methylation in the livers of newborn guinea pigs. Animals received an enteral diet or parenteral nutrition with soybean intravenous lipid emulsion or fish oil containing intravenous lipid emulsion (SMOFlipid, Fresenius Kabi, Bad Homburg, Germany; containing soybean oil, medium chain triglycerides, olive oil, fish oil). Oxidation (redox potential of glutathione) and DNA methylation were higher with parenteral nutrition compared to enteral nutrition. In addition, oxidation and DNA methylation were higher on the fish oil containing mixed lipid emulsion compared to the soybean lipid emulsion.

Allard et al. [107] demonstrated that supplementation of the diet with *n*-3 fatty acids from fish oil (Menhaden oil, National Marine Fisheries Service, Charleston, SC, USA; DHA + EPA = 5.3 g/d) in healthy men resulted in an increase in lipid peroxidation (measured with malondialdehyde (MDA) and lipid peroxides) compared to olive oil supplementation. Lipid peroxidation was not altered by vitamin E supplementation (900 IU/d dL-alpha-tocopherol). Meydani et al. [108] reported increased lipid peroxidation in plasma with fish oil (Pro-Mega, Parke Davis, Detroit, Michigan; 2.4 g DHA + EPA containing 6 IU vitamin E daily) supplementation in healthy women. Brown et al. [109] administered fish oil supplements (MaxEPA, Scherer, Troy, Michigan; 15 g/day) to young men with or without an extra 400 IU vitamin E. Fish oil supplementation increased TBARS (a measure of peroxidation) similarly in both groups. McAnulty et al. [110] administered fish oil supplements (unspecified fish oil from anchovies and sardines; 2.4 g DHA + EPA daily, *n* = 11), a vitamin–mineral mixture (Vitamins C 2000 mg, E 800IU, A 3000IU and selenium 200 µg; *n* = 12), fish oil plus vitamin–mineral mixture (*n* = 13), or placebo (*n* = 12) to healthy individuals for 6 weeks. Lipid peroxidation was assessed with plasma F2-isoprostanes and found to be significantly increased in the fish oil alone group compared to the other groups following exercise. Levels were similar between groups prior to exercise. The increase in F2-isoprostanes with fish oil was attenuated by the vitamin–mineral mixture. However, it should be noted that the amounts of vitamins C and E and selenium administered greatly exceeded recommended dietary intakes for the substances (>33 fold higher for vitamins C and E, 3.6 fold higher for selenium). Rhodes et al. [111] reported increased epidermal lipid peroxidation following ultraviolet irradiation with fish oil supplementation (MaxEPA, Scherer, Troy, Michigan) in human volunteers.

One can also assess the propensity for tissue oxidation following lipid administration by isolating lipoproteins from individuals administered fish oil and other lipids and subjecting the lipoproteins to oxidation. Fish oil supplementation increases *n*-3 PUFA content of circulating lipoproteins. Increased susceptibility of lipoproteins (very low density lipoproteins and low density lipoproteins) to oxidation from fish oil supplemented (SMOFlipid, Fresenius Kabi, Bad Homburg, Germany; Omacor, Reliant Pharmaceuticals, Liberty, NJ, USA; unspecified fish oil) individuals has been shown in a number of studies [112,113,114].

McGrath et al. [115] administered fish oil (MaxEPA, Scherer, Troy, Michigan; 10 g/d; DHA + EPA = 3 g/d; 10 IU alpha-tocopherol) or olive oil to individuals with non-insulin dependent diabetes mellitus in a double-blind randomized cross-over study. Treatment with olive oil did not change plasma MDA or vitamin E levels while treatment with fish oil resulted in elevated MDA and decreased vitamin E levels, compared to baseline and olive oil treatments. Harats et al. [116] administered fish oil (MaxEPA, Scherer, Troy, Michigan; 10 g/day) to smokers and nonsmokers. After 4 weeks of fish oil ingestion, plasma and low density lipoprotein TBARS demonstrated significant increases in smokers and nonsmokers. The rise in TBARS was greater in smokers. Addition of vitamin E (400 mg/d) attenuated, but did not prevent, the increase in TBARS from fish oil. Grundt et al. [117] administered *n*-3 PUFA supplements (Pronova, Oslo, Norway; 3.5 g/d) or corn oil supplements (4 g/d) containing 16 mg/d alpha-tocopherol for 1 year to 255 subjects following myocardial infarction. Serum thiobarbituric acid-malondialdehyde complex levels increased significantly in both groups. However, the increase in the *n*-3 PUFA group was significantly greater than in the corn oil group.

Rice et al. [118] randomized 272 adults with acute lung injury to enteral nutrition supplemented with fish oil, gamma-linolenic acid, and antioxidants (Oxepa, Abbott, Columbus, OH, USA) or an isocaloric control enteral diet. Fish oil supplementation increased plasma EPA levels 8 fold compared to controls. Fish oil supplementation was associated with significantly poorer clinical outcomes (ventilation days, ICU stay, organ failure days, and mortality). F3-isoprostane excretion in the urine, an index of EPA peroxidation, was significantly higher in the fish oil supplemented group. Wu et al. [119] reported superoxide radical and total oxygen radical levels in gastrointestinal surgery patients randomized to a fish oil containing intravenous lipid emulsion (SMOFlipid, Fresenius Kabi, Bad Homburg, Germany; containing soybean oil, medium chain triglycerides (MCT), olive oil, fish oil) or soybean MCT lipid emulsion. Despite superoxide radical levels being lower in the fish oil lipid emulsion group at day 1 (1234 vs. 2004 counts/10 s), maximal levels on day 2 (2260 vs. 1745 counts/10 s) were higher in the fish oil emulsion group. The change in superoxide levels from day 1 to day 6 increased in the fish oil lipid group (+95 counts/10 s) while levels decreased in the soybean MCT emulsion group (−403 counts/10 s). Total oxygen radical levels were comparable in both lipid groups on day 1 (18 counts/10 s soybean MCT vs. 24 counts/10 s mixed fish oil lipid). Levels on day 6 increased in both groups but were much higher in the fish oil containing lipid group (288 vs. 42 counts/10 s). Kosek et al. [120] measured MDA levels and protein carbonyls in long term parenteral nutrition patients receiving fish oil containing lipids. One group received a fish oil lipid containing soybean oil, MCT and fish oil (Lipoplus, B Braun, Melsungen, Germany) and a second group received a lipid emulsion containing soybean oil, MCT, olive oil and fish oil (SMOFlipid, Fresenius Kabi, Bad Homburg, Germany). Both MDA and protein carbonyls were elevated in the parenteral nutrition groups compared to healthy controls. However, despite receiving more fish oil, the group receiving the olive oil containing lipid emulsion had lower levels of these oxidant biomarkers. The investigators concluded that olive oil protected individuals receiving lipid emulsions from oxidative stress. Demirer et al. [121] randomized patients following abdominal surgery to parenteral nutrition with one of three lipid emulsion mixtures: soybean MCT (87.5%, 12.5%), soybean olive (20%, 80%), soybean olive fish (17%, 68%, 15%; fish oil from Omegaven, Fresenius Kabi, Bad Homburg, Germany). TBARS and oxidized low density lipoprotein (LDL)-2 were higher in the soybean olive fish emulsion group and soybean MCT emulsion group (high PUFA formulations) compared to the soybean olive emulsion group. Antebi et al. [112] randomized 20 patients to a mixed soybean MCT olive fish intravenous lipid emulsion (SMOFlipid, Fresenius Kabi, Bad Homburg, Germany) or soybean intravenous lipid emulsion following abdominal surgery. After 5 days of parenteral nutrition with the lipid emulsions, low density lipoproteins were isolated and subjected to in vitro oxidation. Oxidation of the low density lipoproteins from the fish oil containing lipid emulsion group was greater than from the soybean emulsion group. The investigators speculated that *n*-3 PUFA incorporation into low density lipoproteins increased its oxidizability.

It should be acknowledged that not all studies demonstrate an increase in lipid oxidation with fish oil supplementation. Shidfar et al. [122] randomized patients with type 2 diabetes mellitus to *n*-3 fatty acid supplementation (SuperEPA 2000, Advanced Nutritional Technologies, Dublin, California; 1 g DHA + EPA per day) or control lipid supplement (600 mg linoleic acid, 300 mg saturated fatty acids, 100 mg monounsaturated fatty acids) for 10 weeks. There was no significant effects on serum glucose, insulin or hemoglobin A1C levels. Triglycerides decreased 31% in the fish oil group and remained unchanged in the control group. Serum MDA levels decreased in the fish oil group but were unchanged in the control group. In contrast, Toorang et al. [123] studied 81 patients with type 2 diabetes mellitus randomized to *n*-3 PUFA (PBL company, USA; 2.7 g/d) vs. sunflower oil (2.1 g/d; 65% linoleic acid, 12% saturated fatty acids, 23% monounsaturated fatty acids) for two months. There were no differences between groups and no changes from baseline for the anti-oxidant enzymes catalase, glutathione peroxidase, glutathione reductase, and superoxide dismutase (indirect measures of oxidant stress). Linseisen et al. [124] randomized patients undergoing abdominal surgery to a soybean MCT fish intravenous lipid emulsion (Lipoplus, B Braun, Melsungen, Germany) vs. soybean intravenous lipid emulsion. Cholesterol oxidation products (COP) were used as an index of in vivo oxidation. There was no difference between groups and no changes from baseline for COP on Day 0 through Day 6 (during lipid administration).

Overall, the preponderance of data from scientific studies indicates that *n*-3 PUFA supplementation leads to increased lipid peroxidation. Based upon the clinical data, The Dietary Reference Intakes [48] from the Institute of Medicine stated, “Long-chain polyunsaturated fatty acids, particularly DHA and EPA, are vulnerable to lipid peroxidation, resulting in oxidative damage of various tissues.”

It is difficult to compare studies of oxidative stress and to generalize results from one study to the general population of patients due to a variety of uncontrolled variables. Such variables which may affect oxidative stress include lipid dose, type of lipids administered, duration of treatment, underlying disease, inflammatory status of the patients, age, co-morbidities, concomitant administration of other nutrients (i.e., protein, glucose, vitamins, trace elements), and drugs that may possess oxidant or anti-oxidant actions. Failure to protect TPN solutions and lipid emulsions from light has been associated with increased generation of peroxides and lipid peroxidation products [126]. Use of supplemental oxygen and high levels of vitamin E have also been associated with increased oxidation [126]. It is important to note that vitamin E may act as both an anti-oxidant as well as a pro-oxidant (see discussion below). Future studies of oxidative stress should attempt to match patient groups as closely as possible to reduce the impact of these variables.

The relevance of increased lipid peroxidation from use of *n*-3 PUFA supplements requires further study. It is unclear whether such peroxidation is harmful in all patients. It is hypothesized that *n*-3 PUFA induced lipid peroxidation may be beneficial in unstressed individuals by up regulation of anti-oxidant systems while it may exaggerate oxidant damage in patients with severe oxidant stress. In future studies of lipid oxidation, it is important to measure the appropriate endpoints. Ideally, one would utilize a variety of specific lipid peroxidation products as endpoints and relate these end products to tissue integrity markers (i.e., tissue function or damage). In this regard, F3-isoprostanes, derived from EPA oxidation, are a good endpoint for *n*-3 PUFA oxidation while F2-isoprostanes serve as indicators of *n*-6 PUFA oxidation. One could also measure total lipid hydroperoxides in the circulation of tissues or in tissue samples. Removing leukocytes from patients and activating them with various stimulants is not a valid indicator of in vivo lipid peroxidation. When leukocytes activate, they produce reactive oxygen species (ROS). These ROS are indicators of leukocyte function. Suppression of leukocytes by *n*-3 PUFA results in impaired activation and ROS production. This response is not an indicator of lipid peroxidation. For example, Fisher et al. [14] measured stimulated superoxide (opsonized zymosan A) and chemiluminescence (latex beads) responses in monocytes/macrophages from individuals treated with 6 g of DHA/EPA (Cod Liver Oil) per day for 6 weeks. These endpoints reflect upon the oxygen-dependent respiratory burst which promotes the inflammatory response. Both responses were significantly suppressed by *n*-3 PUFA supplementation consistent with the suppressant effect of *n*-3 PUFA upon monocyte functions. The investigators hypothesize that such suppressant effects may be beneficial in patients with atherosclerosis and other inflammatory conditions but may be detrimental in patients with bacterial infections.

In studying lipid peroxidation, it is also important to measure direct products of peroxidation rather than indirect measures of oxidation such as vitamin E levels and levels of anti-oxidant enzymes. Indirect measures of oxidant stress are affected by multiple factors and may not provide an accurate assessment of oxidative stress. This is especially true when administering anti-oxidants such as vitamin E with the lipids. The choice of the control lipid is also important. Control lipids are not truly placebos since they are composed of fatty acids that also have effects upon peroxidation, membrane composition, and immune functions. Since PUFA are more susceptible to peroxidation (due to their multiple double bonds), the use of lipids with low oxidizability (such as saturated fatty acids or monounsaturated fatty acids) may be more appropriate controls. Alternatively, one may want to compare a new lipid to an established lipid.

## 5. Vitamin E and Lipid Peroxidation

Vitamin E is important for protecting PUFA in cellular membranes from peroxidation. The greater the oxidation potential (i.e., the higher the PUFA content), the higher the requirement for vitamin E [97]. However, the protective effects of vitamin E against peroxidation are not complete. Most studies investigating the effects of EPA and DHA supplementation have shown an increase in lipid peroxidation despite amounts of vitamin E that are considered adequate to protect from oxidation [97]. Brown et al. [109] reported significant increases in TBARS in volunteers receiving fish oil supplements (MaxEPA, Scherer, Troy, Michigan; 15 g/d) even in the presence of alpha tocopherol (400 IU/d). Allard et al. [107] studied 80 men randomized to receive fish oil (Menhaden oil, National Marine Fisheries Service, Charleston, SC, USA) or olive oil supplements with or without vitamin E (900 IU/d). In those receiving fish oil, plasma MDA and lipid peroxides increased. Vitamin E supplementation failed to alter lipid peroxidation. Wander et al. [127] administered fish oil (National Institutes of Health Fish Oil Test Material Program, Bethesda, MD, USA; 15 g/d) with 0, 100, 200, or 400 mg/d alpha-tocopherol to post-menopausal women. Urinary TBARS and thiobarbituric-malondialdehyde adduct (TBA-MDA) levels, and plasma TBA-MDA levels increased following fish oil supplementation. Urinary TBARS decreased as alpha-tocopherol intake increased but did not return to baseline levels. Urinary and plasma TBA-MDA levels (a more specific marker of peroxidation) failed to respond to alpha-tocopherol intake and remained significantly above baseline levels. Grundt et al. [117] administered *n*-3 PUFA supplements (Pronova, Oslo, Norway; 3.5 g/d) or corn oil supplements (4 g/d) containing 16 mg/d alpha-tocopherol for 1 year to 255 subjects following myocardial infarction. Serum TBA-MDA complex levels (a measure of lipid peroxidation) increased significantly in both groups, despite the presence of high dose vitamin E (4.0–4.6 mg/g). The increase in peroxidation in the *n*-3 PUFA group was significantly greater than in the corn oil group. Harats et al. [116] administered fish oil (MaxEPA, Scherer, Troy, Michigan; 10 g/day) to smokers and nonsmokers. After 4 weeks of fish oil ingestion, plasma and low density lipoprotein TBARS demonstrated significant increases in smokers and nonsmokers. The rise in TBARS was greater in smokers. Addition of vitamin E (400mg/d) attenuated, but did not prevent, the increase in TBARS from fish oil. Haglund et al. [128] administered fish oil (Eskimo-3 and Inuit-3, Cardinova, Sweden; estimated at 8 g/d) with two doses of vitamin E (0.2 and 1 mg/g lipid). MDA levels increased (122%) in the low dose vitamin E group but remained at baseline levels in the high dose vitamin E group. McAnulty et al. [110] administered fish oil supplements (unspecified fish oil from anchovies and sardines; 2.4 g DHA + EPA) with and without a vitamin–mineral mixture (vitamins C 2000 mg, E 800 IU, A 3000 IU and selenium 200 µg) to healthy volunteers. Lipid peroxidation was significantly increased in the fish oil group following exercise. The increase in lipid peroxidation was attenuated with the vitamin–mineral mixture but remained significantly higher than in the control groups. It should be noted that the amounts of vitamins C and E and selenium administered greatly exceeded recommended dietary intakes for the substances (>33 fold higher for vitamins C and E, 3.6 fold higher for selenium). McGrath et al. [115] administered fish oil (MaxEPA, Scherer, Troy, Michigan; 10 g/d; DHA + EPA = 3 g/d) with 10 IU alpha-tocopherol to individuals with non-insulin dependent diabetes mellitus. Lipid peroxidation was increased with the fish oil vitamin E combination.

Although many studies fail to demonstrate a protective effect of vitamin E upon lipid peroxidation following fish oil supplementation, oxidative damage has been shown to be reduced by consumption of vitamin E in some studies [48]. Overall, the protective effect of vitamin E supplementation upon fish oil oxidation in humans are inconsistent with most studies showing increased lipid peroxidation.

Although vitamin E is well known for its anti-oxidant effects, it is also important to note that vitamin E may have pro-oxidant effects [129,130,131]. Alpha tocopherol scavenges free radicals in hydrophobic environments forming an alpha-tocopheroxyl radical [129]. This radical can undergo a number of reactions. It may interact with a second free radical to form non-radical products (anti-oxidant effect). This reaction is likely when free radicals are being formed at high rates. Under milder oxidative conditions, the alpha-tocopheroxyl radical may interact with a PUFA molecule and oxidize it (pro-oxidant activity). Alternatively, the alpha-tocopheroxyl radical may be recycled back to alpha-tocopherol by an antioxidant such as ascorbate (restoring anti-oxidant activity). The exact effect of alpha-tocopherol upon PUFA oxidation depends upon the site of oxidation (i.e., plasma, lipoprotein molecule, cell membrane), availability of alpha-tocopherol and other anti-oxidants (i.e., ascorbate) at the site of oxidation, and the quantity of free radicals formed.

Bowry et al. [132] evaluated the effect of alpha-tocopherol upon lipid peroxidation in low density lipoprotein molecules. The rate of peroxidation declined as alpha-tocopherol was consumed, was faster in the presence than the absence of alpha-tocopherol, and was accelerated by enrichment of alpha-tocopherol in low density lipoproteins (in vitro or by diet). The investigators concluded that alpha-tocopherol may act as a pro-oxidant for low density lipoproteins. Steger et al. [130] compared lipid peroxidation in two 20% soybean oil intravenous lipid emulsions varying in alpha-tocopherol content (8.8 vs. 156.3 mg/L). The content of other vitamin E homologs was similar. Peroxidation was stimulated by storage at 40 degrees C for 35 days. The lipid emulsion with the higher alpha-tocopherol content demonstrated significantly greater lipid peroxidation (peroxide value increased from 0.01 to 33.6 mmol/L with the high vitamin E content vs. 0.07 to 6.23 mmol/L with the low vitamin E content). The investigators concluded that supplementation of lipid emulsions with more than 160 mg/L alpha-tocopherol leads to a pro-oxidant effect. Jung et al. [131] demonstrated that peroxidation progressively increased in soybean oil lipid emulsion (stored for 6 days at 55 °C) as alpha-tocopherol content increased from 100 ppm to 250, 500, and 1000 ppm. Xu et al. [98] reported greater peroxidation of fish oil intravenous lipid emulsion (Omegaven, Fresenius Kabi, Bad Homburg, Germany) vs. a mixed soybean, MCT, olive, fish oil intravenous lipid emulsion (SMOFlipid, Fresenius Kabi, Bad Homburg, Germany) that may relate to the higher vitamin E content (230 vs. 164 mg/L) of the fish oil emulsion. Winterbone et al. [133] reported that supplementation of alpha-tocopherol (1200 IU/day for 4 weeks) resulted in increased mononuclear oxidative DNA damage following glucose administration in patients with type 2 diabetes. The investigators concluded that alpha-tocopherol possesses pro-oxidant actions.

DHA and EPA are stored in cell membranes where they form an integral part of the structure of the membranes. It is unclear if there is adequate vitamin E within cell membranes during all stages of health and illness to protect the fatty acids from peroxidation. It is also unclear whether administration of extra vitamin E with fish oils improves membrane co-localization of PUFA with vitamin E. High doses of vitamin E could act as a pro-oxidant and increase lipid peroxidation within membranes. There may also be a failure to recycle the alpha-tocopheroxyl radical back to alpha-tocopherol, restoring anti-oxidant activity. Thus, highly unsaturated fatty acids are certainly more vulnerable to lipid peroxidation than fatty acids with lower numbers of double bonds (even in the presence of vitamin E).

## 6. Summary

In summary, the major fatty acids found in fish oil are the long chain *n*-3 polyunsaturated fatty acids DHA and EPA. The primary effects of these *n*-3 PUFA upon the immune system are suppressive. *n*-3 PUFA suppress both the inflammatory response to injury and infection and suppress many functions of immune cells (i.e., macrophages, neutrophils, lymphocytes). In animal models of infection, the effects of *n*-3 PUFA supplementation are mixed with some studies showing detrimental effects while others show beneficial effects. *n*-3 PUFA serve are precursors for resolution molecules. However, the effects of *n*-3 PUFA supplementation upon levels of resolution molecules are inconsistent. The inconsistent effects may result from alterations in metabolic pathways for conversion of *n*-3 PUFA to resolution molecules induced by disease. *n*-3 PUFA increase lipid peroxidation even in the presence of vitamin E. The increased lipid peroxidation has the potential to increase oxidative stress. The risk/benefits of *n*-3 PUFA supplementation should consider these immune effects. Although *n*-3 PUFA supplementation may be beneficial in patients with an exaggerated inflammatory response, they may be harmful in patients with immune suppressive states and hypo-inflammation. Future studies of *n*-3 PUFA supplementation should define the immune status of participants.

## Figures and Tables

**Table 1:** Immune Effects of *n*-3 PUFA.

Study	Cells	Immune Effect
In vivo treatment		
Caughey [10]	Human monocytes	Decreased TNFα, IL-1β, PGE2, TxB2
Endres [11]	Human monocytes and PMN	Decreased IL-1β, TNFα, PGE2, PMN chemotaxis
Lee [12]	Human monocytes and PMN	Decreased LTB4, 5-HETE, PMN adherence and chemotaxis
James [13]	Human monocytes	Decreased TNFα, IL-1β
Fisher [14]	Human monocytes, macrophages	Decreased superoxide and chemiluminescence
Wang [21]	Hen spleen and thymus cells	Decreased lymphocyte proliferation
Yessoufou [22]	Murine T-cells	Decreased T-cell suppressive and migratory functions; decreased chemokine receptors (CCR-4, CXCR-4); decreased selectin; decreased Treg functions
Mayer [23]	Human sepsis	Decreased IL-1β, IL-6, IL-8, TNFα
Furukawa [24]	Human surgery	Decreased IL-6
Mayer [25]	Human monocytes	Decreased TNFα, IL-8, adherence, migration
Gurzell [27]	Mice, B lymphocytes	Increased B lymphocyte IL-6, TNFα, CD40; Increased plasma IL-5, IL-13, IL-9; Increased cecal IgA; no effect of phagocytosis
Rockett [28]	Mice, B and T lymphocytes	Increased B cell activation (CD69, IL-6, TNFα, INFγ); decreased IL-2 from T cells
In vitro treatment		
Soyland [15]	Human T-lymphocytes	Decreased proliferation
Zapata-Gonzalez [16]	Human dendritic cells and monocytes	Decreased MIP-3β chemotaxis; enhanced MIP-1α chemotaxis; decreased lymphocyte proliferation; decreased IL-12, IL-10, IL-6
Ferrante [17]	Human PMN	Decreased random migration and chemotaxis
Tull [18]	Human PMN	Decreased transmigration
Oh [19]	Murine macrophages and adipocytes	Decreased TNFα, IL-6, MCP-1, IL-1β; increased IL-10
Weatherill [20]	Murine dendritic cells, T-cells	Decreased CD86, CD40, CD80, MHCII, IL-6, IL-12, T-cell activation
Calder [26]	Human and rat lymphocytes	Decreased proliferation

PMN = polymorphonuclear leukocytes, TNFα = tumor necrosis factor alpha, IL-1β = interleukin 1 beta, IL-5 = interleukin 5, IL-6 = interleukin 6, IL-9 = interleukin 9, IL-10 = interleukin 10, IL-12 = interleukin 12, INFγ = interferon gamma, PGE2 = prostaglandin E2, TxB2 = thromboxane B2, LTB4 = leukotriene B4, 5-HETE = 5-hydroxyeicosatetraenoic acid, MIP-3β = macrophage inflammatory protein 3β, MIP-1α = macrophage inflammatory protein 1α, MCP-1 = monocyte chemoattractant protein-1; CD40, CD69, CD80, CD86 = cluster of differentiation 40, 69, 80, 86 (co-stimulatory signals for lymphocyte activation); MHCII = major histocompatibility complex II, CCR-4 = CC chemokine receptor 4, CXCR-4 = CXC chemokine receptor 4, Treg = T regulatory cells.

**Table 2:** Effect of *n*-3 PUFA supplementation on infection in animal models.

Study	Animal Model	Findings
Schwerbrock [30]	Mice, influenza virus	Impaired resistance to infection
Cruz-Chamorro [31]	Mice, *Listeria monocytogenes*	Increased dissemination and mortality; decreased splenocyte proliferation
Irons [32]	Mice, *Listeria monocytogenes*	Decreased resistance to infection, increased mortality
Bonilla [33]	Mice, *Mycobacterium tuberculosis*	Increased susceptibility to infection; decreased macrophage TNFα, IL-6, IL-1β
Bonilla [34]	Mice, *Mycobacterium tuberculosis*, in vitro	Decreased macrophage killing and response; Decreased TNFα, IL-6, MCP-1
Zapata-Gonzalez [16]	Human, *Mycobacterium tuberculosis*	Higher infections in Eskimo population
Woodworth [35]	Mice, *Helicobacter hepaticus*	Increased colitis and adenocarcinoma
Peck [36]	Mice, burn plus *Pseudomonas aeruginosa*	Increased mortality
Chang [37]	Mice, *Salmonella typhimurium*	Increased mortality
Bjornsson [38]	Mice, *Klebsiella pneumoniae*	Increased survival
Block [39]	Mice, *Klebsiella pneumoniae* and *Plasmodium berghei*	Increased survival; increased IL-1α,TNFα
Clouva-Molyvdas [40]	Mice; *Pseudomonas aeruginosa* and *Salmonella typhimurium*	No effect on survival
Barton [41]	Rat; abscess with *Staphylococcus aureus* and *Bacteroides fragilus*	No effect on survival

**Table 3:** Effect of *n*-3 PUFA Supplementation upon Levels of Resolution Molecules.

Study	Patients	Supplement	Results
Markworth [54]	healthy	EPA or DPA (1 g/d)	DPA: increased maresin 1; no change resolvin D1 EPA: no change maresin 1, resolvin D1, resolvin E1
Barden [55]	healthy	Fish oil (2.4 g/d EPA + DHA)	Increase: resolvin E1 No change: resolvins E2 & E3, 18R-resolvin E3, resolvins D1 & D2, 17R-resolvin D1
Colas [56]	healthy	EPA + DHA (0.7 g/d) with aspirin	Increased: resolvins D1 & D2, 17-epi-protectin D1, resolvins E2 & E3 Decreased: 17-epi-resolvin D1, resolvins D3 & D6, 17-epi-resolvin D3, resolvin E1 No change: resolvin D5
Norris [57]	healthy	EPA + DHA (900, 1800, 3400 mg/d)	Increased SPM cluster (resolvins D1 & E1, lipoxin B4, aspirin triggered (AT)-LXA4, 18-HEPE, 17-HDHA)
Ramirez [58]	Peripheral artery disease	Fish oil (2.2 g/d EPA + DHA)	Increased: resolvin E1 & E3 No increase: resolvin E2, protectin D1, maresin 1 & 2, resolvins D1 & D2 & D3 & D4 & D5, 17R-resolvin D1, 17R-resolvin D3
Keelan [59]	Pregnant women	Fish oil (3.7 g/d *n*-3 PUFA)	Placenta No increase: resolvin D1, 17R-resolvin D1, resolvin D2, protectin D1 (despite increase in 18-HEPE and 17-HDHA)
Elajami [60]	Coronary artery disease	Fish oil (3.4 g/d DHA + EPA)	Increase: resolvins D6 & E2, AT-resolvin D3, AT-protectin D1, AT-lipoxin B4 Decrease: AT-resolvin D1, resolvin D3, lipoxin B4
Barden [61]	Metabolic syndrome	Fish oil (2.4 g/d EPA + DHA)	Increase: resolvins E1 & E2 & E3 No change: 17-HDHA & 14-HDHA, resolvins D1 & D2, 17R-resolvin D1, protectin D1, maresin 1
Fiala [62]	Cognitive impairment	EPA + DHA (2 g/d)	Variable responses for resolvin D1(increased 6 pts, decreased 4 patients, variable levels 4 pts)
Mas [63]	Chronic kidney disease	Fish oil (3.5 g/d EPA + DHA + DPA)	Increased: resolvin D1 No change: 17R-resolvin D1, resolvin D2
Souza [64]	healthy	18-HEPE + 17-HDHA (1.5, 3.0, 4.5 g/d)	Resolvins and protectins increased at 3.0 and 4.5 g/d; no change with 1.5 g/d
Wang [65]	Alzheimer’s disease	Fish oil (1.7 g/d DHA + 0.6 g/d EPA)	Decreased resolvin D1; no difference for lipoxin A4

EPA = eicosapentaenoic acid; DHA = docosahexaenoic acid; DPA = docosapentaenoic acid.

**Table 4:** Studies of *n*-3 PUFA and Lipid Peroxidation.

Study	Subjects	Results
Xu [98]	Lipid emulsions, in vitro	Higher lipid hydroperoxides with fish oil vs. olive soybean oil emulsion
Watkins [99]	Colon cells	DHA increased oxidation, oleic acid decreased oxidation
Ng [100]	Colon cells	DHA increased oxidation and apoptosis
Fuhrman [101]	Mice	DHA increased oxidation compared to oleic acid, olive oil, linoleic acid, soybean oil
Kubo [102]	Rat	DHA increased liver and kidney peroxidation
Oarada [103]	Mice	Fish oil increased peroxidation in liver and kidneys compared to soybean and olive oils
Song [52,104]	Rat	DHA increased lipid peroxidation in plasma, kidneys, and liver
Lavoie [105]	Guinea pig	Fish oil mixed intravenous lipid emulsion (ILE) increased oxidative stress and apoptosis compared to soybean ILE
Lengo [106]	Guinea pig	Oxidation higher with fish oil containing ILE compared to soybean oil ILE
Allard [107]	Men	Fish oil increased lipid peroxidation compared to olive oil
Meydani [108]	Women	Fish oil increased lipid peroxidation
Brown [109]	Men	Fish oil increased lipid peroxidation
McAnulty [110]	Healthy humans	Fish oil increased lipid peroxidation with exercise
Rhodes [111]	Healthy humans	Fish oil increased epidermal lipid peroxidation after UV irradiation
Antebi [112]	Abdominal surgery	Fish oil increased susceptibility of lipoproteins to oxidation
Suzukawa [113]	Hypertension	Fish oil increased susceptibility of lipoproteins to oxidation
Hau [114]	Hypertriglyceridemia	Fish oil increased susceptibility of lipoproteins to oxidation
McGrath [115]	Noninsulin dependent diabetes mellitus	Fish oil increased lipid peroxidation compared to olive oil
Harats [116]	Smokers and nonsmokers	Fish oil increased lipid peroxidation; greater increase in smokers
Grundt [117]	Myocardial infarction	Fish oil increased lipid peroxidation; greater than corn oil
Rice [118]	Acute lung injury	Fish oil containing enteral nutrition increased lipid peroxidation
Wu [119]	Gastrointestinal surgery	Fish oil ILE had higher oxygen radicals compared to soybean MCT (medium chain triglyceride) emulsion
Kosek [120]	Long term parenteral nutrition	Fish oil containing ILE increased lipid and protein peroxidation
Demirer [121]	Abdominal surgery	High PUFA ILE (fish and soybean oils) had higher lipid peroxidation compared with low PUFA ILE
Shidfar [122]	Type 2 diabetes mellitus	*n*-3 PUFA supplement decreased lipid peroxidation; peroxidation unchanged with linoleic acid, saturated fatty acid, monounsaturated fatty acid supplement
Toorang [123]	Type 2 diabetes mellitus	No effect upon oxidation with *n*-3 PUFA supplement and sunflower oil supplement
Linseisen [124]	Abdominal surgery	No effect upon oxidation with fish oil ILE vs. soybean ILE

## Data Availability

All data used in this review are found in the cited articles.
